# Supplementary material for: The Construction and Exploration of a Comprehensive MicroRNA Centered Regulatory Network in Foxtail Millet (Setaria italica L.)
Source: Front Plant Sci. 2022 May 6;13:848474. doi: 10.3389/fpls.2022.848474 (PMC9121102; doi:10.3389/fpls.2022.848474)
Supplement: Supplementary file 6 [file Data_Sheet_6.zip › Sit-miR172a_Seita.9G380100.1_2294_TPlot.pdf]

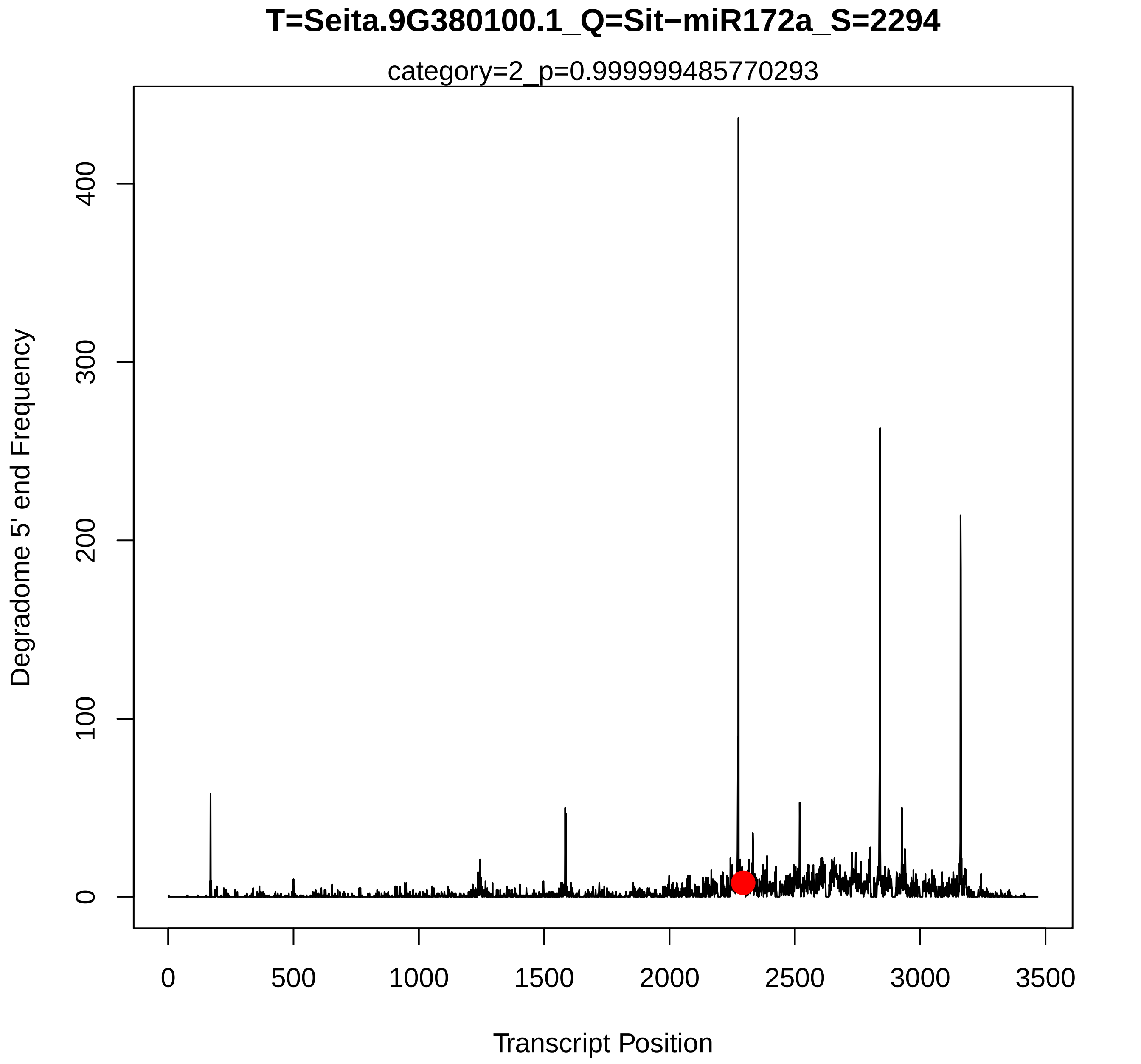

T=Seita.9G380100.1_Q=Sit−miR172a_S=2294
category=2_p=0.999999485770293
Degradome 5' end Frequency
0
100
200
300
400
0
500
1000
1500
2000
2500
3000
3500
Transcript Position
